# [(*Z*)-*N*-(3-Fluoro­phen­yl)-*O*-methyl­thio­carbamato-κ*S*](tri­phenyl­phosphane-κ*P*)gold(I): crystal structure, Hirshfeld surface analysis and computational study

**DOI:** 10.1107/S2056989020009469

**Published:** 2020-07-17

**Authors:** Chien Ing Yeo, Sang Loon Tan, Huey Chong Kwong, Edward R. T. Tiekink

**Affiliations:** a Research Centre for Crystalline Materials, School of Science and Technology, Sunway University, 47500 Bandar Sunway, Selangor Darul Ehsan, Malaysia

**Keywords:** crystal structure, gold, thio­carbamate, Hirshfeld surface analysis, computational chemistry

## Abstract

The title phosphanegold(I) thiol­ate, C_26_H_22_AuFNOPS or [Au(C_8_H_7_FNOS)(C_18_H_15_P)], has the Au^I^ centre coordinated by phosphane-P [2.2494 (8) Å] and thiol­ate-S [2.3007 (8) Å] atoms to define a close to linear geometry [P—Au—S = 176.10 (3)°]. The thiol­ate ligand is orientated so that the meth­oxy-O atom is directed towards the Au atom, forming an Au⋯O close contact of 2.986 (2) Å. In the crystal, a variety of inter­molecular contacts are discerned with fluoro­benzene-C—H⋯O(meth­oxy) and phenyl-C—H⋯F inter­actions leading to dimeric aggregates. These are assembled into a three-dimensional architecture by phenyl-C—H⋯S(thiol­ate) and phenyl-C—H⋯π(fluorobenzene, phen­yl) inter­actions. Accordingly, the analysis of the calculated Hirshfeld surface shows 30.8% of all contacts are of the type C⋯H/H⋯C but this is less than the H⋯H contacts, at 44.9%. Other significant contributions to the surface come from H⋯F/F⋯H [8.1%], H⋯S/S⋯H [6.9%] and H⋯O/O⋯H [3.2%] contacts. Two major stabilization energies have contributions from the phenyl-C—H⋯π(fluoro­benzene) and fluoro­benzene-C—H⋯C(imine) inter­actions (−37.2 kcal mol^−1^), and from the fluoro­benzene-C—H⋯F and phenyl-C—H⋯O inter­actions (−34.9 kcal mol^−1^), the latter leading to the dimeric aggregate.

## Abstract 2

A linear gold-atom geometry defined by phosphane-P and thiol­ate-S atoms is found in the title compound. The packing is stabilized by a combination of fluoro­benzene-C—H⋯O(meth­oxy), phenyl-C—H⋯F, phenyl-C—H⋯S(thiol­ate) and phenyl-C—H⋯π(fluoro­benzene, phen­yl) inter­actions to generate a three-dimensional network.

## Chemical context   

In common with many other phosphanegold(I) thiol­ates (Yeo *et al.*, 2018 ▸), mol­ecules of the general formula *R*_3_PAu[SC(O*R*′)=NAr] have proven to exhibit anti-cancer potential (Ooi *et al.*, 2017 ▸). Complimenting this activity is anti-bacterial potential against Gram-positive bacteria based on *in vitro* assays and time-kill profiles (Yeo *et al.*, 2013 ▸) but not anti-amoebic effects, *i.e*. against *Acanthamoeba castellanii* (Siddiqui *et al.*, 2017 ▸). In keeping with suggestions that the incorporation of fluorine atoms into mol­ecules can enhance their pharmaceutical utility (Müller *et al.*, 2007 ▸; Meanwell, 2018 ▸), it was thought of inter­est to synthesize fluoro analogues of *R*_3_PAu[SC(O*R*′)=NAr].
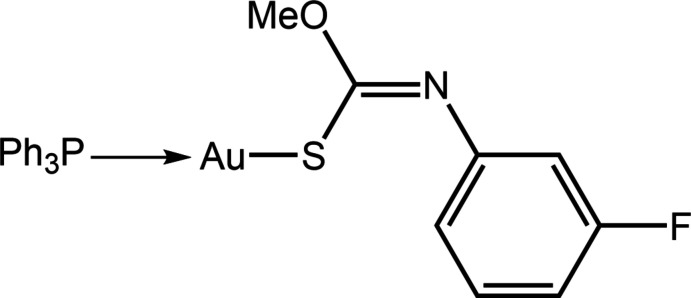


Herein, the compound with *R* = Ph, *R*′ = Me and Ar = 3-fluoro­benzene, (I), is described: synthesis, spectroscopic characterization, crystal structure determination, analysis of the calculated Hirshfeld surfaces and inter­action energies.

## Structural commentary   

**Figure 1:**
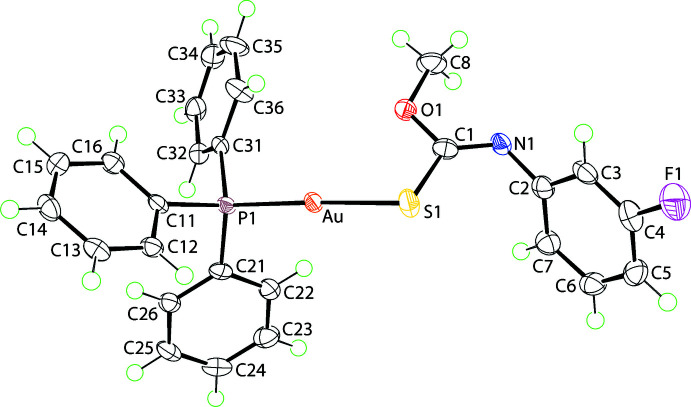
The mol­ecular structures of (I) showing the atom-labelling scheme and displacement ellipsoids at the 70% probability level.

The mol­ecular structure of (I), Fig. 1 ▸, features a linearly coordinated Au^I^ centre defined by phosphane-P1 [2.2494 (8) Å] and thiol­ate-S1 [2.3007 (8) Å] atoms. The deviation of the P1—Au—S1 angle of 176.10 (3)° from the ideal 180° is related to the close approach of the O1 atom, *i.e*. Au⋯O1 = 2.986 (2) Å, as the O1 atom is directed towards the gold atom. The elongation of the C1—S1 bond to 1.762 (3) Å and the shortening of the C1—N1 bond to 1.262 (4) Å with respect to the comparable bonds in the neutral thio­carbamide mol­ecules, *i.e*. S=C(OMe)N(H)Ar (Ho *et al.*, 2005 ▸), *i.e. ca* 1.66 and 1.34 Å, respectively, are consistent with the formation of thiol­ate and imine bonds, respectively.

The overall mol­ecular conformation of (I) is as usually found in mol­ecules formulated as *R*_3_PAu[SC(O*R*′)=NAr]. However, a less common form is known whereby the N-bound aryl ring is orientated towards the gold atom rather than the alk­oxy-oxygen atom (Kuan *et al.*, 2008 ▸). So, rather than an intra­molecular Au⋯O contact, an intra­molecular Au⋯π contact is formed. The observation of both forms in Ph_3_PAu[SC(OEt)=NPh], *i.e*. with Au⋯O (Hall & Tiekink, 1993 ▸) or Au⋯π (Yeo *et al.*, 2016 ▸), suggests the energy difference between the conformations is relatively small. In related binuclear species, DFT calculations suggest that a Au⋯π inter­action is about 6 kcal mol^−1^ more stable than a Au⋯O contact (Yeo *et al.*, 2015 ▸).

## Supra­molecular features   

**Figure 2:**
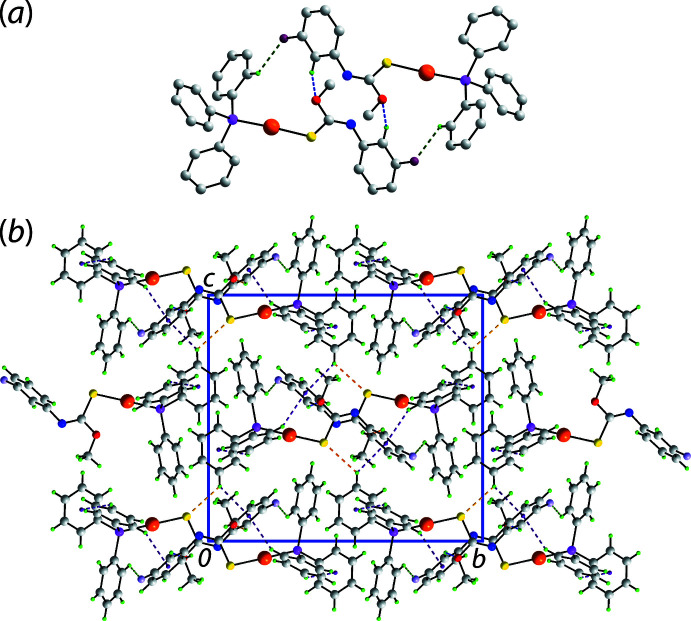
Mol­ecular packing in the crystal of (I): (*a*) the two-mol­ecule aggregate sustained by fluoro­benzene-C—H⋯O and phenyl-C—H⋯F contacts shown as blue and green dashed lines, respectively (non-participating H atoms are omitted) and (*b*) a view of the unit-cell contents down the *a* axis with phenyl-C—H⋯S and phenyl-C—H⋯π(fluoro­benzene, phen­yl) inter­actions shown as orange and purple dashed lines, respectively.

Several directional inter­molecular points of contact between mol­ecules are noted in the extended structure of (I); see Table 1 ▸ for a listing of the geometric parameters characterizing these. Centrosymmetrically related mol­ecules are connected *via* pairwise fluoro­benzene-C—H⋯O1 and phenyl-C—H⋯F1 contacts Fig. 2 ▸(*a*). The dimeric aggregates are connected into a three-dimensional architecture by phenyl-C—H⋯S1 inter­actions, with the phenyl-H atom involved in the latter inter­action, *i.e*. H13, also participating in a C—H⋯π(fluoro­benzene) inter­action and so may be considered bifurcated. The two remaining contacts are of the type phenyl-C—H⋯π(fluoro­benzene, phen­yl) so the fluoro­benzene ring accepts two contacts, one to either side of the ring. A view of the unit-cell contents is shown in Fig. 2 ▸(*b*).

## Hirshfeld surface analysis   

**Figure 3:**
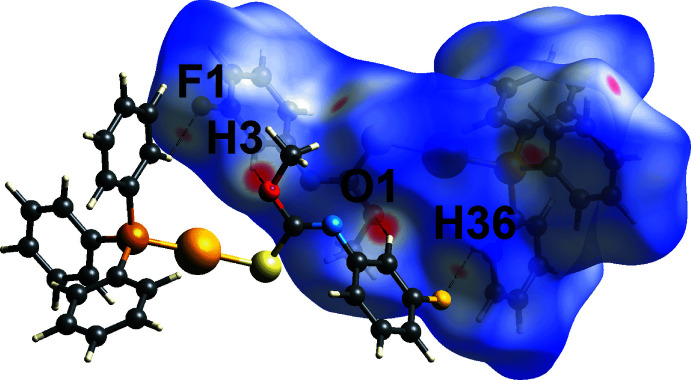
Views of the Hirshfeld surface for (I) mapped over *d*_norm_ in the range −0.222 to +1.382 arbitrary units, highlighting C—H⋯O/F inter­actions.

**Figure 4:**
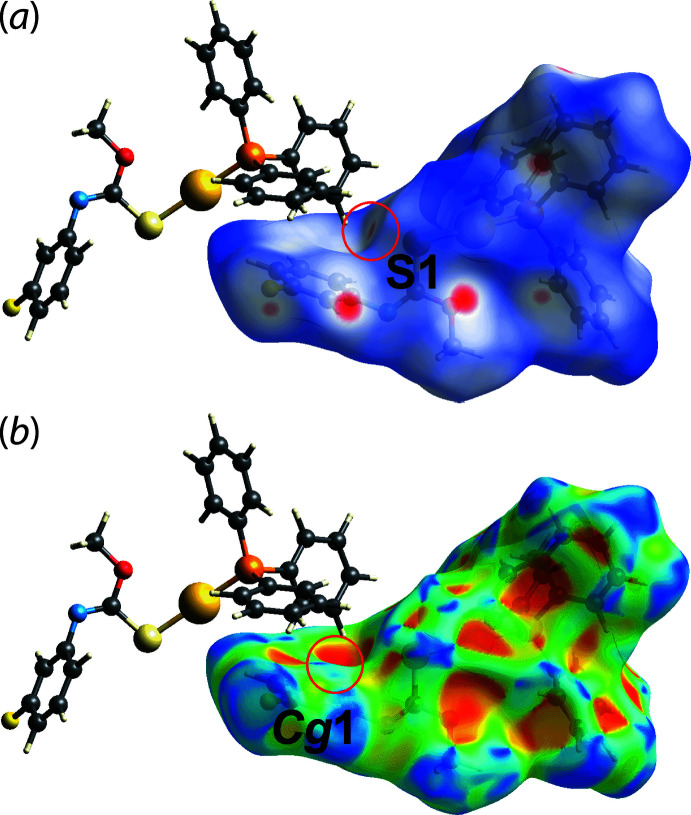
Views of the Hirshfeld surface mapped for (I) over (*a*) *d*_norm_ in the range of −0.222 to +1.382 arbitrary units and (*b*) the shape-index property. The C—H⋯S and C—H⋯π inter­action are highlighted within red circles.

**Figure 5:**
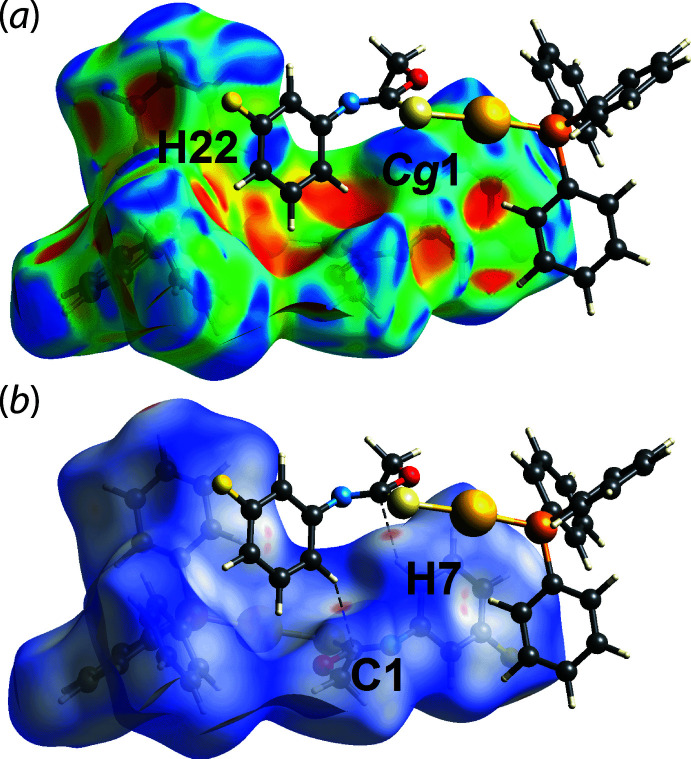
Views of the Hirshfeld surface mapped for (I) over (*a*) the shape-index property and (*b*) *d*_norm_ in the range of −0.222 to +1.382 arbitrary units.

**Figure 6:**
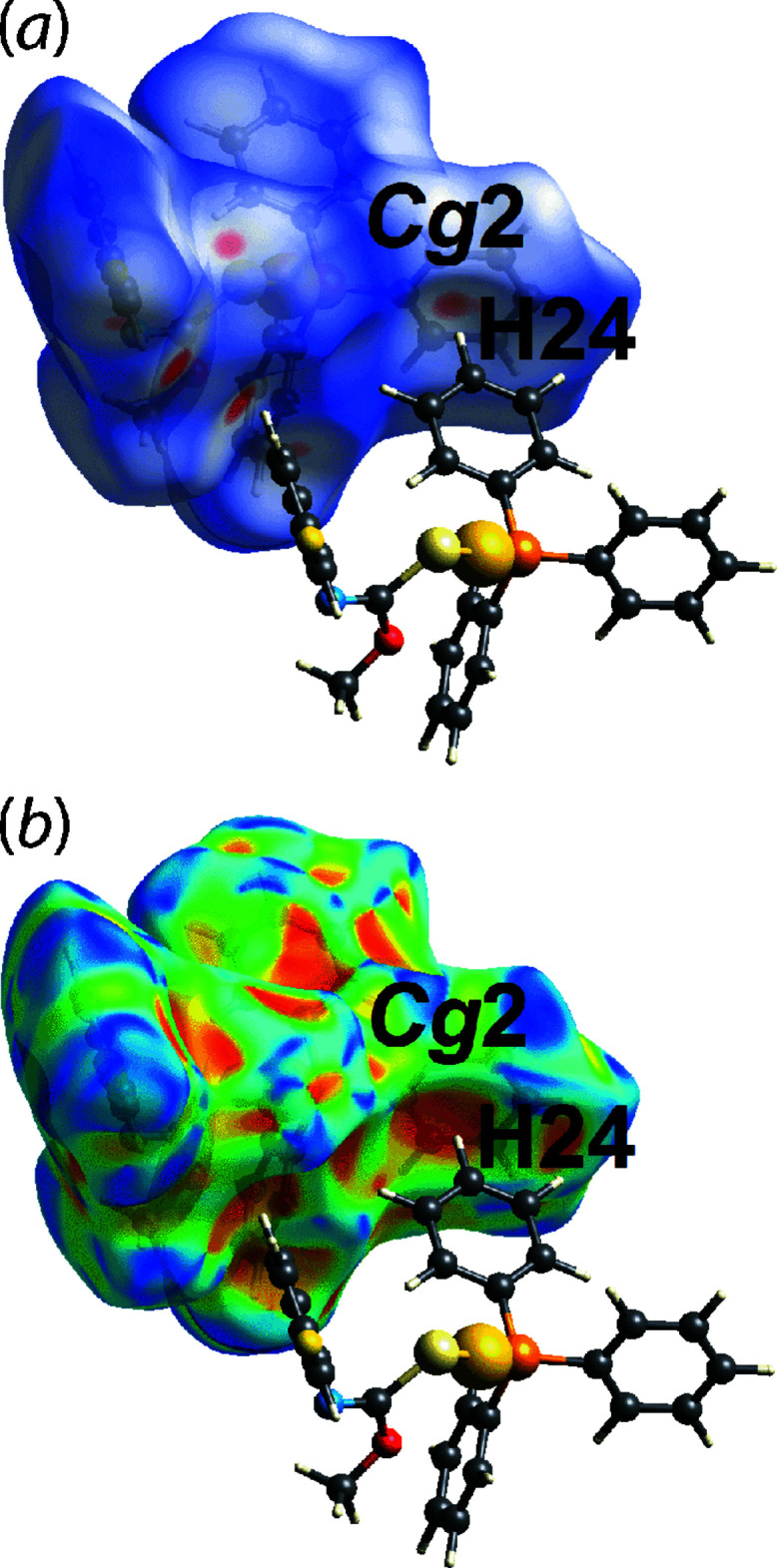
Views of the Hirshfeld surface mapped for (I) over (*a*) *d*_norm_ in the range of −0.222 to +1.382 arbitrary units and (*b*) the shape-index property, each highlighting the phenyl-C24—H24⋯π(C11–C16) inter­action.

**Figure 7:**
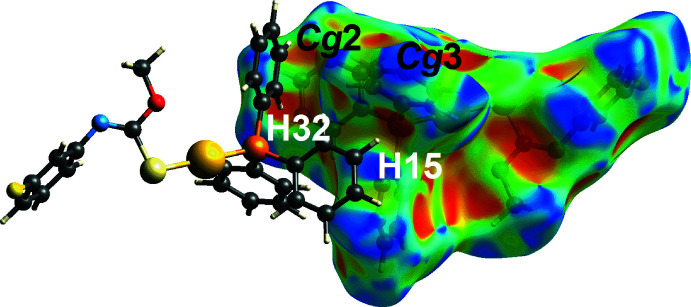
A view of the Hirshfeld surface mapped for (I) over the shape-index property highlighting weak C15—H15⋯π(C21–C26) and C32—H32⋯π(C11–C16) inter­actions.

In order to understand further the inter­actions operating in the mol­ecular packing of (I), the Hirshfeld surfaces mapped over normalized contact distance *d*_norm_ (McKinnon *et al.*, 2004 ▸) and two-dimensional fingerprint plots (Spackman & McKinnon, 2002 ▸) for (I) were generated using *Crystal Explorer 17* (Turner *et al.*, 2017 ▸) following literature procedures (Tan *et al.*, 2019 ▸). The bright-red spots near the fluoro­benzene-H3 and meth­oxy-O1 atoms on the Hirshfeld surface mapped over *d*_norm_ in Fig. 3 ▸, correspond to the fluoro­benzene-C3—H3⋯O1 contacts. These contacts are associated with phenyl-C36—H36⋯F1 contacts, which appear as faint red spots in Fig. 3 ▸, being ∼0.24 Å shorter than the respective sums of their van der Waals radii, Table 2 ▸. The phenyl-C13—H13⋯S1 inter­action is observed as faint red spots on the *d*_norm_ surface in Fig. 4 ▸(*a*), where the cooperative phenyl-C13—H13⋯π(C2–C7) inter­action is shown as a distinctive orange ‘pothole’ on the shape-index-mapped Hirshfeld surface in Fig. 4 ▸(*b*). Although the phenyl-C22—H22⋯π(C2–C7) inter­action was not manifested on the Hirshfeld surface mapped over *d*_norm_, this inter­action shows up as blue ‘bump’ and orange ‘pothole’ near the H22 atom and *Cg*1(C2–C7) centroid, respectively, in Fig. 5 ▸(*a*). Simultaneously, a fluoro­benzene-C7—H7⋯C1(imine) contact, Table 2 ▸, is observed through faint red spots near atoms C1 and H7 on the *d*_norm_ surface in Fig. 5 ▸(*b*). The presence of the phenyl-C24—H24⋯π(C11–C16) contact is evidenced through faint red spots in Fig. 6 ▸(*a*) and the orange ‘pothole’ in Fig. 6 ▸(*b*) on the *d*_norm_ and shape-index mapped Hirshfeld surface, respectively. In addition to the C—H⋯π contacts listed in Table 1 ▸, weak phenyl-C32—H32⋯π(C11–C16) and phenyl-C15—H15⋯π(C21–C26) contacts, Table 2 ▸, are observed as an orange ‘hollow’ on the Hirshfeld surface mapped over shape-index property in Fig. 7 ▸.

**Figure 8:**
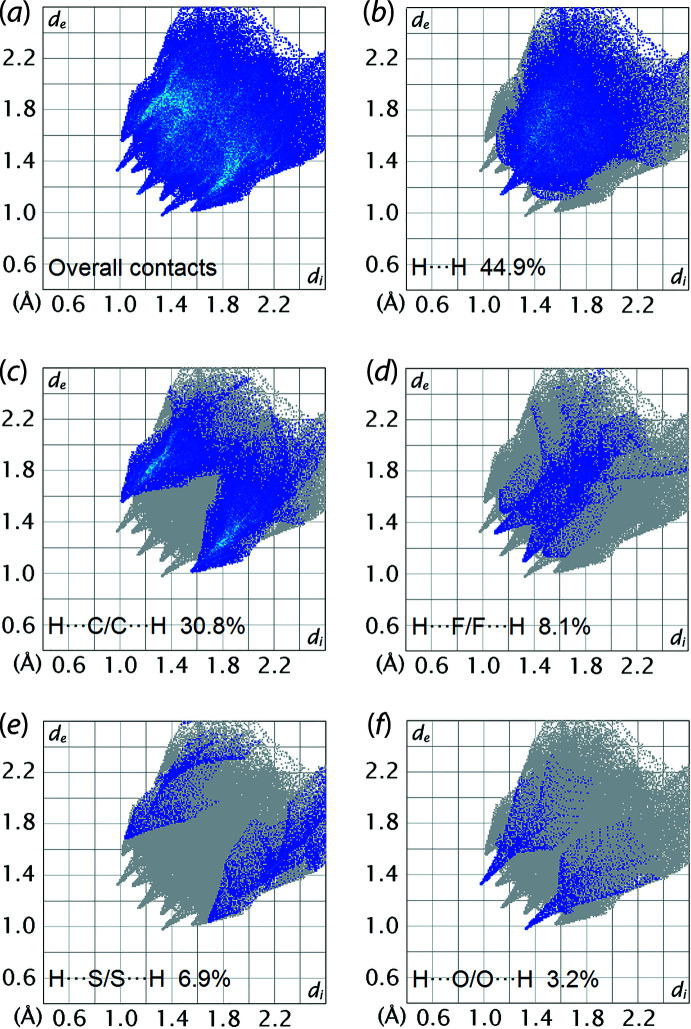
(*a*) The overall two-dimensional fingerprint plots for (I), and those delineated into (*b*) H⋯O/O⋯H, (*c*) H⋯C/C⋯H, (*d*) H⋯F/F⋯H, (*e*) H⋯S/S⋯H and (*f*) H⋯O/O⋯H contacts, with the percentage contributions specified within each plot.

The overall two-dimensional fingerprint plot of (I) is shown in Fig. 8 ▸(*a*), and those delineated into H⋯H, H⋯C/C⋯H, H⋯F/F⋯H, H⋯S/S⋯H and H⋯O/O⋯H contacts are shown in Fig. 8 ▸(*b*)–(*f*), respectively. The percentage contributions for the different inter­atomic contacts to the Hirshfeld surface are summarized in Table 3 ▸. The H⋯H contacts are the most prominent of all contacts and contribute 44.9% to the entire surface. The delineated fingerprint plot in Fig. 8 ▸(*b*) features a beak-shaped peak tipped at *d*_e_ + *d*_i_ ∼2.3 Å. This tip corresponds to a methyl-H8*C*⋯H33(phen­yl) contact and has a distance 0.1 Å shorter than the sum of their van de Waals radii, Table 2 ▸. Consistent with the many C—H⋯π inter­actions evident in the mol­ecular packing, H⋯C/C⋯H contacts contribute 30.8% to the total surface contacts. The H⋯C/C⋯H contacts shows a distinctive feature in the fingerprint plot of Fig. 8 ▸(*c*) with two symmetric spikes at *d*_e_ + *d*_i_ ∼2.4 Å. The tips of pseudo-mirrored sharp spikes at *d*_e_ + *d*_i_ ∼2.4 Å represent the shortest H⋯F/F⋯H contacts (8.1%), Fig. 8 ▸(*d*), and correspond to the phenyl-C36—H36⋯F1 contact in Table 1 ▸. While the C—H⋯O1 and C—H⋯S1 inter­actions are reflected through two sharp-symmetric wings at *d*_e_ + *d*_i_ ∼2.7 and ∼2.5 Å, respectively, Fig. 8 ▸(*e*) and (*f*), these types of contacts only contribute 6.9 and 3.2%, respectively, to the total inter­atomic contacts. The accumulated contribution of the remaining six different inter­atomic contacts is around 6.0% and these do not have a significant influence on the mol­ecular packing.

## Computational chemistry   

The inter­action energies in the crystal of (I) were calculated based on the procedures reported previously (Yusof *et al.*, 2017 ▸). Briefly, the corresponding pairwise mol­ecules were subjected to the calculation *via* the long-range corrected ωB97XD functional combining the D2 version of Grimme’s dispersion model (Chai & Head-Gordon, 2008 ▸), with Pople’s 6-31+G(d,p) basis set (Petersson *et al.*, 1988 ▸; Petersson & Al-Laham, 1991 ▸) comprising the polarization and diffuse functions being employed for C, H, N, O, F, P and S while the effective core potential LANL2DZ (Hay & Wadt, 1985 ▸) was applied for Au. Counterpoise methods (Boys & Bernardi, 1970 ▸; Simon *et al.*, 1996 ▸) were applied to correct for basis set superposition error (BSSE) in the obtained energies. The BSSE corrected inter­action energies (*E*) are listed in Table 4 ▸.

The greatest stabilization energy arises from the phenyl-C22—H22⋯π(C2–C7) and fluoro­benzene-C7—H7⋯C1(imine) inter­actions (−37.2 kcal mol^−1^). This is followed by the phenyl-C36—H36⋯F1 and phenyl-C3—H3⋯O1 inter­actions (−34.9 kcal mol^−1^), which lead to the dimeric aggregate in Fig. 2 ▸(*a*). The other directional contacts outlined in *Supra­molecular features* contribute minor stabilization energies to the mol­ecular packing (−8.9 + −5.46 kcal mol^−1^) while the pairwise weak phenyl-C15—H15⋯π(C21–C26) and phenyl-C32—H32⋯π(C11–C16) inter­actions, which were identified through the *Hirshfeld surface analysis*, have a greater stabilization energy (−15.6 kcal mol^−1^).

## Database survey   

**Figure 9:**
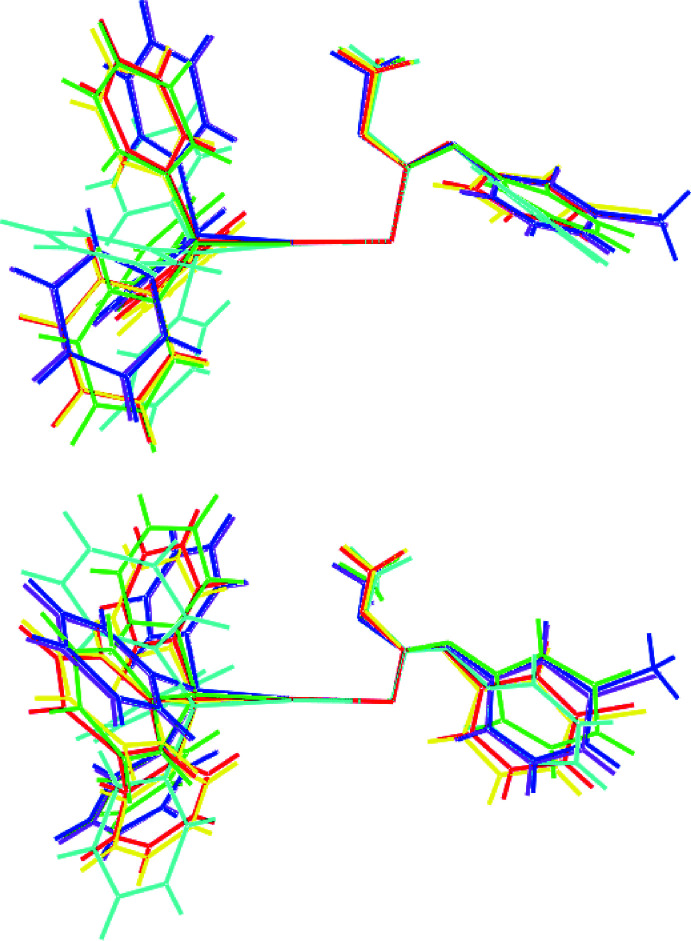
Overlay diagram for (I) (red image) and Ph_3_PAu[SC(OMe)=NC_6_H_4_ *Y*-3] for *Y* = H (green), H (chloro­form solvate, aqua), Me (blue), Cl (triclinic form, pink) and Cl (monoclinic form, yellow). The mol­ecules have been overlapped so the Au, S1 and C1 atoms are coincident.

There are several literature precedents for (I), *i.e*. mol­ecules of the general formula Ph_3_PAu[SC(OMe)=NC_6_H_4_ *Y*-3]. Selected geometric parameters for these are given in Table 5 ▸. To a first approximation, the mol­ecules adopt similar conformations and each features a short intra­molecular Au⋯O inter­action. This being stated, the two overlay diagrams in Fig. 9 ▸ indicate differences in the relative dispositions of the terminal arene rings, as reflected in the differences in the dihedral angles between the planes through the CNOS and C_6_ residues, which vary by up to nearly 15°. Finally, there is an isostructural relationship between (I) and the monoclinic form of the *Y* = Cl compound (Yeo *et al.*, 2016 ▸).

## Synthesis and crystallization   

All chemicals and solvents were used as sourced without further purification. Melting points were determined on a Biobase automatic melting point apparatus MP450 (Jinan, Shandong Province, China). ^1^H and ^13^C{^1^H} NMR spectra were recorded in CDCl_3_ solution on a Bruker Ascend 400 MHz NMR spectrometer (Billerica, MA, USA) with chemical shifts relative to tetra­methyl­silane; the ^31^P{^1^H} NMR spectrum was recorded in CDCl_3_ solution on the same instrument but with the chemical shift recorded relative to 85% aqueous H_3_PO_4_ as the external reference. IR spectra were measured on a Bruker Vertex 70v FTIR spectrophotometer (Billerica, MA, USA) from 4000 to 400 cm^−1^. Elemental analyses were performed on a Leco TruSpec MicroCHN Elemental Analyser (St Joseph, MI, USA).

The thiol precursor, LH, was prepared from the reaction of 3-fluoro­phenyl iso­thio­cyanate (Sigma–Aldrich, St. Louis, MO, USA; 2.50 mmol, 0.38 g) and MeOH (Merck, Kenilworth, NJ, USA; 100 ml) in the presence of NaOH (Merck, Kenilworth, NJ, USA; 2.50 mmol, 0.10 g) followed by the addition of excess 1 *M* HCl. The resulting mixture was extracted using chloro­form, yielding colourless crystals after 3 weeks standing. Yield: 0.421 g (91%), m.p. 334.0–334.5 K. Analysis calculated for C_8_H_8_FNOS: C, 51.88; H, 4.35; N, 7.56%. Found: C, 51.49; H, 4.46; N, 7.42%. IR (cm^−1^): 3241 (*br*) ν(N—H), 1438 (*s*) ν(C—N), 1150 (*s*) ν(C—O), 1048 (*s*) ν (C=S). ^1^H NMR (400 MHz, CDCl_3_, 298 K): δ 8.88 (*s*, *br*, 1H, NH), 7.29–6.87 (*m*, 4H, aryl-H), 4.15 (*s*, 3H, OCH_3_) ppm. ^13^C{^1^H} NMR (400 MHz, CDCl_3_, 298 K): δ 189.4 (Cq), 162.8 (*d*, aryl-C_3_, ^1^ *J*_CF_ = 245.80 Hz), 138.5 (aryl-C_1_), 130.2 (*d*, aryl-C_5_, ^3^ *J*_CF_ = 9.25 Hz), 116.8 (aryl-C_6_), 112.2 (*d*, aryl-C_4_, ^2^ *J*_CF_ = 21.25 Hz), 109.1 (aryl-C_2_), 58.9 (OCH_3_) ppm.

The Ph_3_PAuCl precursor was prepared from the reduction of KAuCl_4_ using sodium sulfite, followed by the addition of a stoichiometric amount of tri­phenyl­phosphane. The precipitate was used as isolated.

NaOH (Merck, Kenilworth, NJ, USA; 0.50 mmol, 0.020 g) in water (5 ml) was added to a suspension of Ph_3_PAuCl (0.50 mmol, 0.247 g) in aceto­nitrile (20 ml), LH (0.50 mmol, 0.093 g) in aceto­nitrile (20 ml) was added and the solution was stirred for 3 h. The solution was left for slow evaporation at room temperature, yielding colourless crystals after 2 weeks. Yield: 0.273 g (85%), m.p. 408.0–408.5 K. Analysis calculated for C_26_H_22_AuFNOPS: C, 48.53; H, 3.45; N, 2.18%. Found: C, 48.73; H, 3.56; N, 1.97%. IR (cm^−1^): 1575 (*s*) ν(C=N), 1122 (*s*) ν(C—O), 1100 (*s*) ν(C—S). ^1^H NMR (400 MHz, CDCl_3_, 298 K): δ 7.55–7.43 (*m*, *br*, 15H, Ph_3_P), 6.95–6.89 (*m*, *br*, 1H, aryl-H_5_), 6.63–6.61 (*m*, *br*, 2H, aryl-H_2,6_), 6.39–6.36 (*m*, *br*, 1H, aryl-H_4_), 3.90 (*s*, 3H, OCH_3_) ppm. ^13^C{^1^H} NMR (400 MHz, CDCl_3_, 298 K): δ 165.4 (Cq), 163.2 (*d*, aryl-C_3_, ^1^ *J*_CF_ = 244.45 Hz), 152.8 (*d*, aryl-C_1_, ^3^ *J*_CF_ = 9.93 Hz), 134.3 (*d*, 2-PC_6_H_5_, ^3^ *J*_CP_ = 13.86 Hz), 131.7 (*d*, 4-PC_6_H_5_, ^4^ *J*_CP_ = 2.30 Hz), 129.7 (*d*, aryl-C_5_, ^3^ *J*_CF_ = 9.62 Hz), 129.3 (*d*, 3-PC_6_H_5_, ^1^ *J*_CP_ = 57.41 Hz), 129.1 (*d*, 2-PC_6_H_5_, ^2^ *J*_CP_ = 11.64 Hz), 117.8 (*d*, aryl-C_6_, ^4^ *J*_CF_ = 2.57 Hz), 109.3 (*d*, aryl-C_2_, ^2^ *J*_CF_ = 21.95 Hz), 109.1 (*d*, aryl-C_4_, ^2^ *J*_CF_ = 21.27 Hz), 55.5 (OCH_3_) ppm. ^31^P{^1^H} NMR (400 MHz, CDCl_3_, 298 K): δ 38.8 ppm.

## Refinement   

Crystal data, data collection and structure refinement details are summarized in Table 6 ▸. The carbon-bound H atoms were placed in calculated positions (C—H = 0.95–0.98 Å) and were included in the refinement in the riding-model approximation, with *U*_iso_(H) set to 1.2–1.5*U*_eq_(C). The maximum and minimum electron density peaks of 1.17 and 1.22 e Å^−3^, respectively, are located 0.97 and 0.69 Å, respectively, from the Au atom.

## Supplementary Material

Crystal structure: contains datablock(s) I, global. DOI: 10.1107/S2056989020009469/hb7932sup1.cif

Structure factors: contains datablock(s) I. DOI: 10.1107/S2056989020009469/hb7932Isup2.hkl

CCDC reference: 2015568

Additional supporting information:  crystallographic information; 3D view; checkCIF report

## Figures and Tables

**Table 1:** Hydrogen-bond geometry (Å, °) *Cg*1 and *Cg*2 are the centroids of the (C2–C7) and (C11–C16) rings, respectively.

*D*—H⋯*A*	*D*—H	H⋯*A*	*D*⋯*A*	*D*—H⋯*A*
C3—H3⋯O1^i^	0.95	2.42	3.269 (3)	148
C36—H36⋯F1^i^	0.95	2.51	3.218 (4)	131
C13—H13⋯S1^ii^	0.95	2.83	3.519 (3)	130
C13—H13⋯*Cg*1^ii^	0.95	2.74	3.500 (3)	137
C22—H22⋯*Cg*1^iii^	0.95	2.63	3.397 (3)	138
C24—H24⋯*Cg*2^iv^	0.95	2.80	3.552 (3)	137

Symmetry codes: (i) 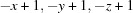; (ii) 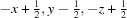; (iii) 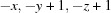; (iv) 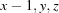.

**Table 2:** A summary of short inter­atomic contacts (Å) for (I)^*a*^

Contact	Distance	Symmetry operation
C3—H3⋯O1^*b*^	2.31	−*x* + 1, −*y* + 1, −*z* + 1
C36—H36⋯F1^*b*^	2.43	−*x* + 1, −*y* + 1, −*z* + 1
C13—H13⋯S1^*b*^	2.74	−*x* + 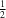 , *y* − 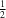 , −*z* + 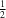
C7—H7⋯C1	2.66	−*x*, −*y* + 1, −*z* + 1
H8*C*⋯H33	2.31	−*x* + 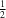 , *y* + 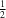 , −*z* + 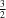
C15—H15⋯*Cg*(C21–C26)	3.23	−*x*, −*y*, −*z* + 1
C32—H32⋯*Cg*(C11–C16)	3.22	−*x*, −*y*, −*z* + 1

Notes: (*a*) The inter­atomic distances are calculated in *Crystal Explorer 17* (Turner *et al.*, 2017 ▸) whereby the *X*—H bond lengths are adjusted to their neutron values; (*b*) these inter­actions correspond to those discussed above in *Supra­molecular features*.

**Table 3:** Percentage contributions of inter­atomic contacts to the Hirshfeld surface for (I)

Contact	Percentage contribution
H⋯H	44.9
H⋯C/C⋯H	30.8
H⋯F/F⋯H	8.1
H⋯S/S⋯H	6.9
H⋯O/O⋯H	3.2
Others	6.1

**Table 4:** A summary of inter­action energies (kcal mol^−1^) calculated for (I)

Contact	*E* ^BSSE^_int_	Symmetry operation
C22—H22⋯π(C2–C7) (×2) +		
C7—H7⋯C1 (×2)	−37.2	−*x*, −*y* + 1, −*z* + 1
C36—H36⋯F1 (×2) +		
C3—H3⋯O1 (×2)	−34.9	−*x* + 1, −*y* + 1, −*z* + 1
C32—H32⋯π(C11–C16) (×2) +		
C15—H15⋯π(C21–C26) (×2)	−15.6	−*x*, −*y*, −*z* + 1
C13—H13⋯π(C2–C7) +		
C13—H13⋯S1	−8.9	−*x* + 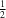 , *y* − 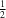 , −*z* + 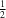
C24—H24⋯π(C11–C16)	−5.4	*x* − 1, *y*, *z*

**Table 5:** A summary of key geometric parameters (Å, °) for structures related to (I)

*Y*	Au—S	Au—P	P—Au—S	Au⋯O	CNOS/C_6_	REFCODE	Ref.
H	2.3005 (14)	2.2578 (12)	177.72 (4)	3.045 (4)	87.18 (18)	HADZAN	Hall & Tiekink (1993 ▸)
H^*a*^	2.3102 (14)	2.2613 (12)	175.96 (5)	3.140 (3)	78.4 (2)	COCRUI	Kuan *et al.* (2008 ▸)
Me	2.2968 (15)	2.2479 (11)	175.12 (4)	2.954 (3)	74.69 (16)	COCROC	Kuan *et al.* (2008 ▸)
Cl^*b*^	2.2903 (17)	2.2416 (14)	174.61 (5)	2.988 (3)	75.01 (14)	VUYKOQ	Tadbuppa & Tiekink (2010 ▸)
Cl^*c*^	2.3071 (15)	2.2535 (15)	175.62 (5)	3.052 (3)	73.95 (16)	VUYKOQ01	Yeo *et al.* (2016 ▸)
F	2.3007 (8)	2.2494 (8)	176.10 (3)	2.986 (2)	78.73 (9)	–	This work

Notes: (*a*) chloro­form solvate; (*b*) *P* 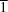 polymorph; (*c*) *P*2_1_/*c* polymorph.

**Table 6:** Experimental details

Crystal data	
Chemical formula	[Au(C_8_H_7_FNOS)(C_18_H_15_P)]
*M*_r_	643.44
Crystal system, space group	Monoclinic, *P*2_1_/*n*
Temperature (K)	100
*a*, *b*, *c* (Å)	8.9311 (3), 17.2458 (6), 15.6857 (5)
β (°)	99.361 (3)
*V* (Å^3^)	2383.80 (14)
*Z*	4
Radiation type	Mo *K*α
μ (mm^−1^)	6.35
Crystal size (mm)	0.30 × 0.30 × 0.30

Data collection	
Diffractometer	Agilent Technologies SuperNova Dual diffractometer with Atlas detector
Absorption correction	Multi-scan (*CrysAlis PRO*; Agilent, 2013 ▸)
*T*_min_, *T*_max_	0.252, 1.000
No. of measured, independent and observed [*I* > 2σ(*I*)] reflections	25800, 5429, 5017
*R*_int_	0.045
(sin θ/λ)_max_ (Å^−1^)	0.650

Refinement	
*R*[*F* ^2^ > 2σ(*F* ^2^)], *wR*(*F* ^2^), *S*	0.023, 0.050, 1.08
No. of reflections	5429
No. of parameters	290
H-atom treatment	H-atom parameters constrained
Δρ_max_, Δρ_min_ (e Å^−3^)	1.17, −1.22

Computer programs: *CrysAlis PRO* (Agilent, 2013 ▸), *SHELXS97* (Sheldrick, 2015*a* ▸), *SHELXL2018/3* (Sheldrick, 2015*b* ▸), *ORTEP-3 for Windows* (Farrugia, 2012 ▸), *DIAMOND* (Brandenburg, 2006 ▸) and *publCIF* (Westrip, 2010 ▸).

## supplementary crystallographic information

### Crystal data

**Table:**

[Au(C_8_H_7_FNOS)(C_18_H_15_P)]	*F*(000) = 1248
*M**_r_* = 643.44	*D*_x_ = 1.793 Mg m^−^^3^
Monoclinic, *P*2_1_/*n*	Mo *K*α radiation, λ = 0.71073 Å
*a* = 8.9311 (3) Å	Cell parameters from 12325 reflections
*b* = 17.2458 (6) Å	θ = 3.9–29.4°
*c* = 15.6857 (5) Å	µ = 6.35 mm^−^^1^
β = 99.361 (3)°	*T* = 100 K
*V* = 2383.80 (14) Å^3^	Block, colourless
*Z* = 4	0.30 × 0.30 × 0.30 mm

### Data collection

**Table:**

Agilent Technologies SuperNova Dual diffractometer with Atlas detector	5429 independent reflections
Radiation source: SuperNova (Mo) X-ray Source	5017 reflections with *I* > 2σ(*I*)
Mirror monochromator	*R*_int_ = 0.045
Detector resolution: 10.4041 pixels mm^-1^	θ_max_ = 27.5°, θ_min_ = 2.8°
ω scan	*h* = −11→11
Absorption correction: multi-scan (CrysAlis PRO; Agilent, 2013)	*k* = −19→22
*T*_min_ = 0.252, *T*_max_ = 1.000	*l* = −20→20
25800 measured reflections	

### Refinement

**Table:**

Refinement on *F*^2^	Primary atom site location: structure-invariant direct methods
Least-squares matrix: full	Secondary atom site location: difference Fourier map
*R*[*F*^2^ > 2σ(*F*^2^)] = 0.023	Hydrogen site location: inferred from neighbouring sites
*wR*(*F*^2^) = 0.050	H-atom parameters constrained
*S* = 1.08	*w* = 1/[σ^2^(*F*_o_^2^) + (0.019*P*)^2^ + 0.9042*P*] where *P* = (*F*_o_^2^ + 2*F*_c_^2^)/3
5429 reflections	(Δ/σ)_max_ = 0.002
290 parameters	Δρ_max_ = 1.17 e Å^−^^3^
0 restraints	Δρ_min_ = −1.22 e Å^−^^3^

### Special details

**Table:**

Geometry. All esds (except the esd in the dihedral angle between two l.s. planes) are estimated using the full covariance matrix. The cell esds are taken into account individually in the estimation of esds in distances, angles and torsion angles; correlations between esds in cell parameters are only used when they are defined by crystal symmetry. An approximate (isotropic) treatment of cell esds is used for estimating esds involving l.s. planes.

### Fractional atomic coordinates and isotropic or equivalent isotropic displacement parameters (Å^2^)

**Table:**

	*x*	*y*	*z*	*U*_iso_*/*U*_eq_	
Au	0.19089 (2)	0.29511 (2)	0.43142 (2)	0.01422 (5)	
S1	0.25242 (9)	0.42032 (5)	0.40015 (4)	0.02170 (17)	
P1	0.11477 (8)	0.17558 (4)	0.46267 (4)	0.01214 (15)	
F1	0.3783 (2)	0.74491 (12)	0.34520 (12)	0.0342 (5)	
O1	0.3182 (2)	0.40907 (13)	0.56743 (11)	0.0174 (4)	
N1	0.2716 (3)	0.53368 (14)	0.52214 (14)	0.0166 (5)	
C1	0.2802 (3)	0.46274 (18)	0.50373 (17)	0.0166 (6)	
C2	0.2227 (3)	0.58863 (18)	0.45714 (17)	0.0168 (6)	
C3	0.3275 (3)	0.63954 (18)	0.43078 (18)	0.0196 (6)	
H3	0.432629	0.635322	0.453168	0.023*	
C4	0.2749 (4)	0.69595 (18)	0.3716 (2)	0.0220 (7)	
C5	0.1236 (4)	0.70522 (18)	0.3365 (2)	0.0224 (7)	
H5	0.091705	0.744606	0.295236	0.027*	
C6	0.0213 (3)	0.65520 (19)	0.36383 (18)	0.0214 (7)	
H6	−0.083664	0.660487	0.341475	0.026*	
C7	0.0685 (3)	0.59706 (18)	0.42351 (18)	0.0200 (6)	
H7	−0.003907	0.562999	0.441510	0.024*	
C8	0.3549 (4)	0.4400 (2)	0.65366 (18)	0.0259 (7)	
H8A	0.382956	0.397437	0.694599	0.039*	
H8B	0.266609	0.467333	0.668620	0.039*	
H8C	0.440246	0.476108	0.656412	0.039*	
C11	0.2043 (3)	0.09645 (17)	0.41333 (16)	0.0137 (6)	
C12	0.2005 (3)	0.09928 (18)	0.32403 (16)	0.0157 (6)	
H12	0.154329	0.141957	0.291578	0.019*	
C13	0.2641 (3)	0.03972 (18)	0.28306 (18)	0.0186 (6)	
H13	0.261179	0.041530	0.222276	0.022*	
C14	0.3322 (3)	−0.02258 (18)	0.32988 (18)	0.0197 (6)	
H14	0.375062	−0.063543	0.301276	0.024*	
C15	0.3376 (3)	−0.02491 (18)	0.41910 (18)	0.0198 (6)	
H15	0.384366	−0.067488	0.451488	0.024*	
C16	0.2747 (3)	0.03502 (18)	0.46069 (17)	0.0165 (6)	
H16	0.279928	0.033867	0.521660	0.020*	
C21	−0.0875 (3)	0.16265 (17)	0.42616 (16)	0.0139 (6)	
C22	−0.1844 (3)	0.22366 (19)	0.43861 (18)	0.0190 (6)	
H22	−0.144129	0.270054	0.465862	0.023*	
C23	−0.3395 (4)	0.2166 (2)	0.4112 (2)	0.0233 (7)	
H23	−0.405616	0.257836	0.420261	0.028*	
C24	−0.3976 (3)	0.1491 (2)	0.37058 (18)	0.0220 (7)	
H24	−0.503754	0.144248	0.351765	0.026*	
C25	−0.3024 (3)	0.08884 (19)	0.35728 (17)	0.0197 (6)	
H25	−0.343172	0.043087	0.328736	0.024*	
C26	−0.1474 (3)	0.09489 (18)	0.38545 (16)	0.0161 (6)	
H26	−0.082202	0.053035	0.377065	0.019*	
C31	0.1488 (3)	0.15679 (17)	0.57834 (16)	0.0140 (6)	
C32	0.0449 (3)	0.11807 (17)	0.61907 (17)	0.0177 (6)	
H32	−0.047892	0.100392	0.586516	0.021*	
C33	0.0759 (3)	0.10496 (18)	0.70764 (17)	0.0206 (6)	
H33	0.004161	0.078454	0.735568	0.025*	
C34	0.2111 (4)	0.13046 (19)	0.75517 (18)	0.0255 (7)	
H34	0.232732	0.121070	0.815605	0.031*	
C35	0.3144 (4)	0.1695 (2)	0.71456 (19)	0.0274 (8)	
H35	0.407258	0.186851	0.747336	0.033*	
C36	0.2843 (4)	0.1838 (2)	0.62661 (19)	0.0232 (7)	
H36	0.355046	0.211765	0.599282	0.028*	

### Atomic displacement parameters (Å^2^)

**Table:**

	*U*^11^	*U*^22^	*U*^33^	*U*^12^	*U*^13^	*U*^23^
Au	0.01968 (7)	0.01105 (8)	0.01111 (7)	−0.00252 (4)	0.00000 (4)	−0.00008 (4)
S1	0.0393 (5)	0.0135 (4)	0.0121 (3)	−0.0044 (3)	0.0039 (3)	−0.0002 (3)
P1	0.0144 (3)	0.0121 (4)	0.0093 (3)	−0.0020 (3)	0.0002 (3)	−0.0002 (3)
F1	0.0338 (11)	0.0293 (12)	0.0403 (11)	−0.0116 (9)	0.0089 (9)	0.0050 (9)
O1	0.0231 (11)	0.0158 (12)	0.0122 (10)	0.0007 (8)	−0.0002 (8)	−0.0017 (8)
N1	0.0174 (12)	0.0141 (14)	0.0176 (12)	0.0003 (10)	0.0005 (9)	−0.0050 (10)
C1	0.0123 (13)	0.0215 (18)	0.0161 (14)	−0.0016 (12)	0.0024 (11)	−0.0005 (12)
C2	0.0187 (14)	0.0165 (17)	0.0154 (13)	0.0013 (12)	0.0029 (11)	−0.0048 (12)
C3	0.0174 (15)	0.0193 (18)	0.0208 (15)	−0.0017 (12)	−0.0001 (12)	−0.0085 (13)
C4	0.0259 (17)	0.0181 (18)	0.0239 (16)	−0.0059 (13)	0.0097 (13)	−0.0038 (13)
C5	0.0287 (18)	0.0176 (18)	0.0201 (16)	0.0058 (13)	0.0018 (13)	−0.0018 (12)
C6	0.0168 (15)	0.0229 (19)	0.0231 (15)	0.0057 (13)	−0.0009 (12)	−0.0043 (13)
C7	0.0159 (15)	0.0204 (18)	0.0238 (15)	−0.0006 (12)	0.0039 (12)	−0.0021 (13)
C8	0.0304 (18)	0.032 (2)	0.0130 (14)	0.0037 (15)	−0.0039 (12)	−0.0030 (13)
C11	0.0150 (14)	0.0132 (16)	0.0121 (12)	−0.0042 (11)	−0.0002 (10)	−0.0028 (11)
C12	0.0176 (14)	0.0169 (17)	0.0119 (13)	−0.0014 (12)	0.0000 (10)	−0.0010 (11)
C13	0.0193 (15)	0.0225 (18)	0.0137 (13)	−0.0038 (12)	0.0022 (11)	−0.0046 (12)
C14	0.0171 (15)	0.0193 (18)	0.0233 (15)	−0.0007 (12)	0.0050 (12)	−0.0091 (13)
C15	0.0202 (15)	0.0161 (17)	0.0219 (15)	0.0021 (12)	−0.0002 (12)	0.0015 (12)
C16	0.0182 (14)	0.0179 (17)	0.0135 (13)	−0.0018 (12)	0.0026 (11)	−0.0013 (12)
C21	0.0155 (14)	0.0192 (17)	0.0068 (12)	−0.0026 (11)	0.0013 (10)	0.0020 (11)
C22	0.0192 (15)	0.0182 (17)	0.0186 (15)	0.0017 (12)	−0.0002 (12)	−0.0039 (12)
C23	0.0192 (15)	0.027 (2)	0.0227 (15)	0.0059 (13)	0.0008 (12)	−0.0003 (14)
C24	0.0157 (15)	0.034 (2)	0.0151 (14)	−0.0024 (13)	−0.0010 (11)	0.0014 (13)
C25	0.0227 (16)	0.0212 (18)	0.0141 (14)	−0.0094 (13)	−0.0007 (11)	−0.0019 (12)
C26	0.0174 (14)	0.0169 (17)	0.0135 (13)	−0.0018 (12)	0.0007 (11)	0.0000 (12)
C31	0.0186 (14)	0.0114 (16)	0.0111 (13)	0.0008 (11)	−0.0006 (10)	−0.0028 (11)
C32	0.0202 (15)	0.0146 (16)	0.0175 (14)	0.0008 (12)	0.0006 (11)	−0.0005 (12)
C33	0.0312 (17)	0.0159 (17)	0.0153 (14)	−0.0003 (13)	0.0051 (12)	0.0028 (12)
C34	0.042 (2)	0.0215 (19)	0.0117 (14)	0.0042 (15)	−0.0012 (13)	0.0003 (13)
C35	0.0310 (18)	0.031 (2)	0.0163 (15)	−0.0063 (15)	−0.0088 (13)	−0.0016 (14)
C36	0.0239 (16)	0.0272 (19)	0.0181 (15)	−0.0089 (14)	0.0017 (12)	−0.0048 (14)

### Geometric parameters (Å, º)

**Table:**

Au—P1	2.2494 (8)	C13—H13	0.9500
Au—S1	2.3007 (8)	C14—C15	1.393 (4)
S1—C1	1.762 (3)	C14—H14	0.9500
P1—C11	1.817 (3)	C15—C16	1.389 (4)
P1—C21	1.818 (3)	C15—H15	0.9500
P1—C31	1.819 (3)	C16—H16	0.9500
F1—C4	1.365 (3)	C21—C26	1.396 (4)
O1—C1	1.364 (3)	C21—C22	1.396 (4)
O1—C8	1.441 (3)	C22—C23	1.387 (4)
N1—C1	1.262 (4)	C22—H22	0.9500
N1—C2	1.408 (4)	C23—C24	1.387 (5)
C2—C3	1.394 (4)	C23—H23	0.9500
C2—C7	1.400 (4)	C24—C25	1.379 (4)
C3—C4	1.374 (4)	C24—H24	0.9500
C3—H3	0.9500	C25—C26	1.387 (4)
C4—C5	1.382 (4)	C25—H25	0.9500
C5—C6	1.375 (4)	C26—H26	0.9500
C5—H5	0.9500	C31—C32	1.382 (4)
C6—C7	1.390 (4)	C31—C36	1.399 (4)
C6—H6	0.9500	C32—C33	1.390 (4)
C7—H7	0.9500	C32—H32	0.9500
C8—H8A	0.9800	C33—C34	1.384 (4)
C8—H8B	0.9800	C33—H33	0.9500
C8—H8C	0.9800	C34—C35	1.379 (5)
C11—C16	1.385 (4)	C34—H34	0.9500
C11—C12	1.397 (4)	C35—C36	1.384 (4)
C12—C13	1.382 (4)	C35—H35	0.9500
C12—H12	0.9500	C36—H36	0.9500
C13—C14	1.385 (4)		
			
P1—Au—S1	176.10 (3)	C13—C14—C15	119.7 (3)
C1—S1—Au	101.30 (10)	C13—C14—H14	120.1
C11—P1—C21	104.91 (13)	C15—C14—H14	120.1
C11—P1—C31	106.09 (13)	C16—C15—C14	120.0 (3)
C21—P1—C31	106.78 (12)	C16—C15—H15	120.0
C11—P1—Au	115.21 (9)	C14—C15—H15	120.0
C21—P1—Au	111.34 (10)	C11—C16—C15	120.0 (2)
C31—P1—Au	111.91 (10)	C11—C16—H16	120.0
C1—O1—C8	115.4 (2)	C15—C16—H16	120.0
C1—N1—C2	120.6 (2)	C26—C21—C22	119.7 (3)
N1—C1—O1	120.5 (2)	C26—C21—P1	122.3 (2)
N1—C1—S1	127.5 (2)	C22—C21—P1	118.1 (2)
O1—C1—S1	112.0 (2)	C23—C22—C21	120.1 (3)
C3—C2—C7	119.3 (3)	C23—C22—H22	120.0
C3—C2—N1	119.6 (3)	C21—C22—H22	120.0
C7—C2—N1	120.8 (3)	C22—C23—C24	119.7 (3)
C4—C3—C2	118.3 (3)	C22—C23—H23	120.1
C4—C3—H3	120.9	C24—C23—H23	120.1
C2—C3—H3	120.9	C25—C24—C23	120.5 (3)
F1—C4—C3	118.0 (3)	C25—C24—H24	119.7
F1—C4—C5	118.3 (3)	C23—C24—H24	119.7
C3—C4—C5	123.7 (3)	C24—C25—C26	120.2 (3)
C6—C5—C4	117.4 (3)	C24—C25—H25	119.9
C6—C5—H5	121.3	C26—C25—H25	119.9
C4—C5—H5	121.3	C25—C26—C21	119.8 (3)
C5—C6—C7	121.2 (3)	C25—C26—H26	120.1
C5—C6—H6	119.4	C21—C26—H26	120.1
C7—C6—H6	119.4	C32—C31—C36	119.9 (2)
C6—C7—C2	120.1 (3)	C32—C31—P1	122.1 (2)
C6—C7—H7	120.0	C36—C31—P1	118.0 (2)
C2—C7—H7	120.0	C31—C32—C33	120.1 (3)
O1—C8—H8A	109.5	C31—C32—H32	120.0
O1—C8—H8B	109.5	C33—C32—H32	120.0
H8A—C8—H8B	109.5	C34—C33—C32	120.1 (3)
O1—C8—H8C	109.5	C34—C33—H33	120.0
H8A—C8—H8C	109.5	C32—C33—H33	120.0
H8B—C8—H8C	109.5	C35—C34—C33	119.8 (3)
C16—C11—C12	120.0 (3)	C35—C34—H34	120.1
C16—C11—P1	122.6 (2)	C33—C34—H34	120.1
C12—C11—P1	117.4 (2)	C34—C35—C36	120.8 (3)
C13—C12—C11	119.7 (3)	C34—C35—H35	119.6
C13—C12—H12	120.1	C36—C35—H35	119.6
C11—C12—H12	120.1	C35—C36—C31	119.4 (3)
C12—C13—C14	120.6 (3)	C35—C36—H36	120.3
C12—C13—H13	119.7	C31—C36—H36	120.3
C14—C13—H13	119.7		
			
C2—N1—C1—O1	−175.8 (2)	P1—C11—C16—C15	178.3 (2)
C2—N1—C1—S1	5.8 (4)	C14—C15—C16—C11	1.1 (4)
C8—O1—C1—N1	−3.5 (4)	C11—P1—C21—C26	12.2 (2)
C8—O1—C1—S1	175.15 (19)	C31—P1—C21—C26	−100.1 (2)
Au—S1—C1—N1	−157.0 (2)	Au—P1—C21—C26	137.4 (2)
Au—S1—C1—O1	24.52 (19)	C11—P1—C21—C22	−166.8 (2)
C1—N1—C2—C3	−107.2 (3)	C31—P1—C21—C22	80.9 (2)
C1—N1—C2—C7	78.0 (4)	Au—P1—C21—C22	−41.6 (2)
C7—C2—C3—C4	−0.7 (4)	C26—C21—C22—C23	0.5 (4)
N1—C2—C3—C4	−175.6 (3)	P1—C21—C22—C23	179.5 (2)
C2—C3—C4—F1	−179.1 (2)	C21—C22—C23—C24	−0.7 (5)
C2—C3—C4—C5	0.0 (4)	C22—C23—C24—C25	0.1 (5)
F1—C4—C5—C6	179.8 (3)	C23—C24—C25—C26	0.8 (4)
C3—C4—C5—C6	0.7 (5)	C24—C25—C26—C21	−1.0 (4)
C4—C5—C6—C7	−0.8 (4)	C22—C21—C26—C25	0.4 (4)
C5—C6—C7—C2	0.1 (4)	P1—C21—C26—C25	−178.6 (2)
C3—C2—C7—C6	0.6 (4)	C11—P1—C31—C32	−94.4 (3)
N1—C2—C7—C6	175.5 (3)	C21—P1—C31—C32	17.1 (3)
C21—P1—C11—C16	−109.9 (2)	Au—P1—C31—C32	139.2 (2)
C31—P1—C11—C16	2.9 (3)	C11—P1—C31—C36	86.5 (3)
Au—P1—C11—C16	127.3 (2)	C21—P1—C31—C36	−161.9 (2)
C21—P1—C11—C12	70.2 (2)	Au—P1—C31—C36	−39.9 (3)
C31—P1—C11—C12	−177.0 (2)	C36—C31—C32—C33	−1.0 (4)
Au—P1—C11—C12	−52.6 (2)	P1—C31—C32—C33	179.9 (2)
C16—C11—C12—C13	1.5 (4)	C31—C32—C33—C34	−0.2 (4)
P1—C11—C12—C13	−178.7 (2)	C32—C33—C34—C35	0.6 (5)
C11—C12—C13—C14	−0.3 (4)	C33—C34—C35—C36	0.2 (5)
C12—C13—C14—C15	−0.5 (4)	C34—C35—C36—C31	−1.3 (5)
C13—C14—C15—C16	0.1 (4)	C32—C31—C36—C35	1.8 (5)
C12—C11—C16—C15	−1.9 (4)	P1—C31—C36—C35	−179.1 (3)

### Hydrogen-bond geometry (Å, º)

*Cg*1 and *Cg*2 are the centroids of the (C2–C7) and (C11–C16) rings, respectively.

**Table:**

*D*—H···*A*	*D*—H	H···*A*	*D*···*A*	*D*—H···*A*
C3—H3···O1^i^	0.95	2.42	3.269 (3)	148
C36—H36···F1^i^	0.95	2.51	3.218 (4)	131
C13—H13···S1^ii^	0.95	2.83	3.519 (3)	130
C13—H13···*Cg*1^ii^	0.95	2.74	3.500 (3)	137
C22—H22···*Cg*1^iii^	0.95	2.63	3.397 (3)	138
C24—H24···*Cg*2^iv^	0.95	2.80	3.552 (3)	137

Symmetry codes: (i) −*x*+1, −*y*+1, −*z*+1; (ii) −*x*+1/2, *y*−1/2, −*z*+1/2; (iii) −*x*, −*y*+1, −*z*+1; (iv) *x*−1, *y*, *z*.
